# Comparison of interobserver agreement between the evaluation of bicipital and the patellar tendon reflex in healthy dogs

**DOI:** 10.1371/journal.pone.0219171

**Published:** 2019-07-10

**Authors:** Felix Giebels, Laura Pieper, Barbara Kohn, Holger Andreas Volk, Nadia Shihab, Shenja Loderstedt

**Affiliations:** 1 Small Animal Clinic (WE20), Department of Veterinary Medicine, Freie Universität Berlin, Berlin, Germany; 2 Department of Clinical Veterinary Medicine, Division of Clinical Neurology, Vetsuisse Faculty of Bern, University of Bern, Bern, Switzerland; 3 Department of Veterinary Medicine, Institute for Veterinary Epidemiology and Biostatistics, Freie Universität Berlin, Berlin, Germany; 4 Department of Small Animal Medicine and Surgery, University of Veterinary Medicine Hannover, Hannover, Germany; 5 Southern Counties Veterinary Specialists, Department of Neurology and Neurosurgery, Forest Corner Farm, Hangersley, Ringwood, Hampshire, United Kingdom; 6 Small Animal Department, Neurology and Neurosurgery Unit, College of Veterinary Medicine, University of Leipzig, Leipzig, Germany; University of Witwatersrand, SOUTH AFRICA

## Abstract

The reliability of reflex-assessment is currently debatable, with current literature regarding the patellar tendon reflex (PTR) as highly reliable, while the biceps tendon reflex (BTR) is regarded to be of low reliability in the dog. Such statements are, however, based on subjective observations rather than on an empirical study. The goals of this study were three-fold: (1) the quantification of the interobserver agreement (IA) on the evaluation of the canine bicipital (BTR) and patellar tendon (PTR) reflex in healthy dogs, (2) to compare the IA of the BTR and PTR evaluation and (3) the identification of intrinsic (*sex*, *age*, *fur length*, *weight*) and extrinsic (*observer´s expertise*, *body side*) risk factors on the IA of both reflexes. The observers were subdivided into three groups based on their expected level of expertise (neurologists = highest -, practitioners = middle–and veterinary students = lowest level of expertise). For the BTR, 54 thoracic limbs were analyzed and compared to the evaluation of the PTR on 64 pelvic limbs. Each observer had to evaluate the reflex presence (RP) (*present* or *absent*) and the reflex activity (RA) using a 5-point ordinal scale. Multiple reliability coefficients were calculated. The influence of the risk factors has been calculated using a mixed regression-model. The Odds Ratio for each factor was presented. The higher the level of expertise the higher was the IA of the BTR. For RP(BTR), IA was highest for neurologists and for RA(BTR) the IA was lowest for students. The level of expertise had a significant impact on the degree of the IA in the evaluation of the bicipital tendon reflex: for the RA(BTR), practitioners had a 3.4-times (p = 0.003) and students a 7.0-times (p < 0.001) higher chance of discordance. In longhaired dogs the chance of disagreement was 2.6-times higher compared to shorthaired dogs in the evaluation of RA(BTR) (p = 0.003). Likewise, the IA of the RP(PTR) was the higher the higher the observers´ expertise was with neurologists having significantly highest values (p < 0.001). The RA(PTR) has been evaluated more consistent by practitioners and students than the RA(BTR). For practitioners this difference was significant (< 0.01). Our data suggests that neurologists assess the bicipital and patellar tendon reflex in dogs most reliably. None of the examined risk factors had a significant impact on the degree of IA in the evaluation of RP(PTR), while students had a 4.4-times higher chance of discordance when evaluating the RA(PTR) compared to the other groups. This effect was significant (p < 0.001). Neurologists can reliably assess the bicipital and patellar tendon reflex in healthy dogs. Observer´s level of expertise and the fur length of the dog affect the degree of IA of RA(BTR). The influence of the observer´s expertise is higher on the evaluation of the BTR than on the PTR.

## Introduction

The evaluation of the reflex answer of different segmental reflexes is fundamental in the examination of the neurological patient [1–3]. Reflex assessment can be used in neuroanatomical localization of a lesion and for monitoring disease progression in a patient with neurological dysfunction. The bicipital reflex is often used in human medicine in the assessment of the integrity of the upper limb´s reflex arc [4–6]. However, assessment of reflexes in the daily clinical setting can be highly subjective [1, 7, 8] and has the potential to be influenced by various factors including the age of the patient [9–11], the muscle temperature [12, 13], the observer´s level of expertise [7, 14, 15] or the examination itself [16, 17]. Interestingly, different studies have shown that both the degree of reliability and sensitivity are variable [7, 18–22]. Considering the clinical importance of case discussion and communication between different practitioners their clinical examination findings need to be comparable.

Different segmental spinal reflexes in thoracic and pelvic limbs are described in the veterinary literature [1, 3, 23–25]. The assessment of some of the reflexes are thought to have a high degree of reliability (e. g. the flexor reflex or the patellar tendon reflex), whilst others are depicted to be of low reliability (e. g. the biceps or triceps tendon reflex) [1, 3, 23, 25, 26]. Difficulty in eliciting the reflex or the often assumed low sensitivity are reasons for a postulated low reliability [23, 25, 26].

The aims of this study were three- fold: (1) to evaluate if the reflex answer of the biceps tendon (BTR) and the patellar tendon reflex (PTR) in healthy dogs can be reliably assessed, (2) to compare the IA of the BTR and PTR evaluation and (3) to identify intrinsic and extrinsic factors, that influence level of the IA.

## Material and methods

### Selection and subdivision of dogs

Dogs that did not have any history of neurological disease and in which general clinical and neurological examinations were performed prior to reflex evaluation were included. All examined thoracic and pelvic limbs were divided into two groups based on each of the following factors: the dog´s age, sex, weight, fur length and body side. The categories' cut-off values were chosen based on the median value of each parameter (Table 1).

**Table 1:** Categorization of examined intrinsic risk factors.

Risk factor	subdivision	criteria
Age	young	< 6 years
	old	≥ 6 years
Sex	female	female
	male	male
Weight	light	≤ 20 kg
	heavy	> 20 kg
Fur length	shorthaired	< 8 cm
	longhaired	≥ 8 cm
Body side	left	left limb
	right	right limb

### Procedure

All dogs examined during this study have been presented to the Small Animal Clinic (WE20), Department of Veterinary Medicine, Freie Universität Berlin, Berlin, Germany as patients between September 2012 until July 2015. All examinations were performed in the same room. The examination and reflex evaluation was part of the clinically required neurological examination. The only difference to a “routine” neurological examination was the fact, that the reflex evaluation was videotaped. The procedure was explained to the owners who gave consent for their dogs to participate in the study, and who were present at the time of the examination. The ethics statement committee of the Department of Veterinary Medicine, Freie Universität Berlin did therefore approve this study (faculty representative: Prof. Barbara Kohn, DVM).

The examinations were videotaped using a HD-camera (HDR-FX7E, Sony, Japan). The camera was mounted on a stand in a fixed position at a height of 110cm and with an angle of view of 30° in relation to the ground. All examinations were performed in a standardized manner by the same examiner (FG) with the dog in lateral recumbency and the examined limb on the upper side [24]. The owner was positioned at the dog´s head, calming the patient. The camera was equipped with an autofocus and automatic white balance so that the quality of the recordings was maintained independent of the fur colour and of slight movements in the examined limb. Light conditions were standardized within the room through artificial illumination. Each limb was assigned a randomized number between 1 and 100 and anonymized.

### Video processing

Two separate video recordings were prepared: one for the BTR (study 1) and one for the PTR (study 2). The individual examination clips contributing to each recording were cut using Windows Movie Maker (Version 2012, Microsoft Corporation) in ascending order, with a video clip of each limb comprising ten hits with the reflex hammer. The mean duration of the video clips was 13.71 (8.57–34.63) seconds for the BTR- and 7.76 (5.4–10.03) seconds for the PTR. The entire video recording length after processing was 19:02 minutes for the BTR- and 11:42 minutes for the PTR-tape. Both recordings were saved in mp4-format and forwarded to the observers via Dropbox or Youtube.

### Observers and evaluation

Nine observers evaluated both video recordings. The observers were subdivided dependent on their expected level of expertise, into three groups of three observers each. The first group was comprised of three (HV, NS, SL) board-certified neurologists (ECVN) (N1-N3) and was expected to have the highest level of expertise. The second group, which was rated as the group with the medium level of expertise, included three small animal veterinary practitioners (P1-P3) without a specialisation in veterinary neurology, but with two to three years’ experience working in small animal practice. The lowest level of expertise was expected for the third group, which consisted of three final-year veterinary students (S1–S3 Tables).

All observers evaluated the video sequences separately from each other and were blinded to the identity and to the history of the examined dogs. For each examination video clip, observers had to assess the reflex-presence (*reflex present*; *reflex absent*) and the degree of reflex-activity using a previously described 5-point-ordinal scale (*0 = absent; 1 = reduced; 2-normal; 3 = increased; 4 = clonic*) [24].

### Statistical analysis

Different reliability coefficients were calculated to assess the IA of each group. For each pair of observers within one group Kappa analysis and the percentage agreement (r%) was calculated (S1–S4 Tables), resulting in three values for each coefficient and for each group. For the reflex-presence, Cohen´s Kappa (K_C_) and for the reflex-activity, the weighted Kappa (K_w_) was calculated. The group´s IA was determined using the mean r% ($\bar{X}r\%$), the mean K_C_ ($\bar{X}K_{C}$) or K_w_ ($\bar{X}K_{w}$) respectively, Fleiss-Kappa (K_F Pres_ and K_F Akt_) and the intraclass correlation coefficient (ICC). All coefficients were calculated for both the reflex-presence and -activity, but since K_F_ doesn´t weight the level of disagreement, the ICC revealed the more reliable result for the group´s IA of reflex-activity. According to Stam and van Crevel [22] all reflex-activity evaluations of each group were categorized depending on their level of agreement as depicted in Table 2.

**Table 2:** Definition of the level of agreement [22].

Full agreement	all 3 observers evaluate identically
Partial (dis)agreement (1 point)	one observer evaluates differently with 1 scale-point
Partial (dis)agreement (≥ 2 points)	one observer evaluates differently with at least 2 scale-points
Complete disagreement	all 3 observers evaluate differently

All Kappa-coefficients were interpreted following Landis and Koch [27] with *<0*.*00 = poor; 0*.*00–0*.*20 = slight; 0*.*21–0*.*40 = fair; 0*.*41–0*.*60 = moderate; 0*.*61–0*.*80 = substantial; 0*.*81–1*.*00 = almost perfect to perfect*. The ICC was interpreted following Vincent and Weir [28] who defines an ICC > 0.90 as *high*, between 0.80 and 0.90 as *moderate* and between 0.70 and 0.80 as *questionable* reliability. In doing so, values < 0.70 will be ignored as to be of *poor* reliability.

The difference of K_F_ and ICC between groups and both reflexes was interpreted as significant, if there was no overlap of the respective 95% confidence interval (CI95%) and the K_F_-, or ICC-value of the compared group, respectively. According to recommendations from Burn and Weir [29], Kappa was presented together with its respective interpretation-parameters (S1–S4 Tables). In doing so, the Prevalence-Index (PI), which quantifies the homogeneity of the evaluations, the Bias-Index (BI), that depicts the symmetry of the evaluations and the maximum Kappa (K_max_) of each K_C_- and K_w_-value, that defines the maximum possible value of Kappa-agreement, were presented. Additionally, following Burn and Weir [29], the *clinical acceptance* of the calculated K-value for each pair of observers was categorized (Table 3).

**Table 3:** Definition of *clinical acceptance* [29].

category	%	K_C_ / K_w_	interpretation
**I.**	75	≥ 0.40	clinically acceptable
**II.**	75	0.00–1.00	clinically non-acceptable
**III.**	75	< 0.40	clinically inconclusive

Univariable and multivariable regression-analyses were conducted to evaluate the impact of the risk factors *age*, *sex*, *weight*, *fur length*, *body side*, and *observer´s level of expertise* on reflex-presence and -activity (BTR and PTR) agreement among observers within each group. Therefore, agreement was categorized as *1* if there was complete agreement among the three observers of each group and as *0* if there was partial or complete disagreement. Mixed logistic regression modelling was used to account for repetition of assessments on the same legs. After univariable analyses, risk factors with a liberal p-value < 0.10 were selected for building a multivariable model. The strength of the effect was presented as Odds Ratio (OR) with p < 0.05 indicating significant impact.

## Results

Thirty dogs passed the inclusion criteria for the BTR-assessment. The dogs had a median age of 5.8 (1–14) years and a median weight of 17.5 (5.8–57) kg. In one dog, the right thoracic limb was amputated. After inspecting the video footage, the examinations of two right and one left thoracic limbs were excluded from evaluation due to excitability and excessive movement of the examined dog, resulting in 56 sequences for BTR evaluation of 30 dogs.

For the PTR assessment, 64 pelvic limbs of 32 dogs were included. The included dogs had a median age of 6.4 (0.8–11.0) years and a median weight of 25.5 (2.0–45.0) kg. The categorisation of the examined limbs into groups is displayed in Table 4 for both the BTR and the PTR studies.

**Table 4:** Subdivision of the examined thoracic and pelvic limbs.

category	BTR		PTR	
	N	%	N	%
**sex**				
male	23	41.1	20	31.1
Female	33	58.9	44	68.8
Total:	56	100.0	64	100.0
**Age (years)**				
<6	26	46.4	32	50.0
≥6	30	53.6	32	50.0
Total:	56	100.0	64	100.0
**weight (kg)**				
<20	34	60.7	26	40.6
≥20	22	39.3	38	59.4
Total:	56	100.0	64	100.0
**fur length**				
shorthaired	33	58.9	46	71.9
longhaired	23	41.1	18	28.1
Total:	56	100.0	64	100.0
**body side**				
right	27	48.2	32	50.0
left	29	51.8	32	50.0
Total:	56	100.0	64	100.0

### Reflex-presence

All reliability coefficients are tabulated in Table 5. The higher the level of the observer´s expertise the higher was the IA for reflex-presence (BTR). Cohen´s Kappa was interpreted as *clinically acceptable* for the reflex-presence (BTR)-evaluations of all pairs of observers. The level of expertise had a significant impact on K_F_ values for the reflex-presence (BTR) with the lowest agreement observed for students (0.45; CI95%: 0.303–0.606, p < 0.001). Fleiss´ Kappa ranged from *moderate* (students) to *substantial* (neurologists) for the reflex presence (BTR). The ICC (BTR) was highest for the neurologists (0.91; CI95%: 0.859–0.944), who reached a *high* IA and lowest for students (*questionable*: 0.73; CI95%: 0.576–0.832). This difference was significant (p < 0.001).

**Table 5:** Results of reliability analysis for the reflex presence.

	${\bar{X}}_{r\%}$	$\bar{X}K_{C}$	K_F Pres_	SE	CI95%		ICC	CI95%	
					lower	upper		lower	upper
**Biceps tendon reflex**									
**Neurologists**	97.6	0.76	0.77^a^	0.077	0.61	0.91	0.91^a^	0.86	0.94
**Practicioners**	95.2	0.63	0.64^a^	0.077	0.49	0.79	0.85^b^	0.77	0.91
**Students**	91.7	0.47	0.45^b^	0.077	0.30	0.61	0.73^c^	0.58	0.83
**Patellar tendon reflex**									
**Neurologists**	97.9	0.43	0.49^a^	0.072	0.35	0.63	0.74^a^	0.61	0.84
**Practicioners**	97.9	0.22	0.32^b^	0.072	0.18	0.46	0.60^a^^,^^b^	0.39	0.74
**Students**	89.6	0.25	0.23^b^	0.072	0.09	0.37	0.50^b^	0.25	0.68

The coefficients are the higher the higher the observer´s level of expertise.

${\bar{X}}_{r\%}$, mean percentage agreement between the three observer pairs of each group; $\bar{X}K_{C}$, mean K_C_ between the three observer pairs of each group; K_F Pres_, Fleiss´ Kappa for the reflex presence with its standard error (SE) and the lower and upper 95% confidence interval (CI95%) values; ICC, intraclass correlation coefficient with its CI95% values.

^a,b,c^, different letters indicate significant differences at p < 0.05.

The IA of reflex-presence (K_F Pres_, ICC) for the PTR also increased with the observer´s expertise. Students had the lowest IA for the evaluation of the reflex-presence (PTR) for all reliability coefficients. Cohen´s Kappa was interpreted as *clinically acceptable* in the neurologists-group for two, for practitioners for one and for students for none of the pairs of observers, when evaluating reflex-presence (PTR) (S2 Table). Fleiss’ Kappa for the neurologists was interpreted as *moderate* (0.49; CI95%: 0.348–0.631) and it was significantly higher than for the practitioners (p = 0.02) and students (p < 0.001) (both *fair*). Nevertheless, the $\bar{X}r\%$ was nearly 98% for neurologists and practitioners for the assessment of the PTR reflex presence. The ICC decreases with decrease of the observer’s expertise.

For the reflex-presence analysis, K_F Pres_, $\bar{X}K_{C}$ and ICC were lower for the PTR compared to the BTR within each group. In contrast, $\bar{X}r\%$ was slightly higher for the PTR for the neurologists and practitioners but lower for students compared to the reflex-presence of the BTR.

In the univariable regression analysis of the reflex presence (BTR), students had greater odds of judging discordantly when compared to neurologists (OR = 4.337; CI95%: 0.795–23.654) (Table 6), nevertheless, this difference was not significant (p = 0.09). The other factors did not influence judgement (p > 0.05). For the reflex-presence (PTR), a tendency for students (p = 0.054) to have greater odds for discordant judgement (OR = 3.8; CI95%: 0.976–14.497) could be calculated (Table 7). Other factors did not influence the assessment of reflex-presence (PTR).

**Table 6:** Results of univariable mixed regression analysis for the BTR-evaluation.

Risk factor	Reflex-presence				Reflex-activity			
	Odds Ratio (OR)	CI 95% (OR)		p-value	Odds Ratio (OR)	CI 95% (OR)		p-value
		lower	upper			lower	upper	
**Body side**								
right	Reference	.	.		Reference	.	.	
left	1.27	0.30	5.41	0.741	1.05	0.56	1.95	0.888
**Sex**								
male	Reference	.	.		Reference	.	.	
female	1.28	0.29	5.61	0.745	0.94	0.50	1.76	0.835
**Fur length**								
shorthaired	Reference				Reference			
Longhaired	1.38	0.33	5.84	0.659	2.65	1.39	5.02	0.003*
**Weight (kg)**								
<20	Reference	.	.		Reference	.	.	
≥20	0.54	0.11	2.60	0.443	1.25	0.66	2.36	0.492
**Age (years)**								
<6	Reference	.	.		Reference	.	.	
≥6	0.80	0.19	3.39	0.762	0.99	0.53	1.85	0.983
**Observer**								
neurologists	Reference	.	.		Reference	.	.	
Practitioners	1.58	0.23	10.59	0.639	3.43	1.54	7.66	0.003*
students	4.34	0.80	23.65	0.090	6.98	3.02	16.13	<0.001*

* = significant

**Table 7:** Results of univariable mixed regression analysis for the PTR-evaluation.

Risk factor	Reflex-presence				Reflex-activity			
	Odds Ratio (OR)	CI 95% (OR)		p-value	Odds Ratio (OR)	CI 95% (OR)		p-value
		lower	upper			lower	upper	
**Body side**								
right	Reference	.	.		Reference	.	.	
left	1.67	0.53	5.23	0.376	1.73	0.97	3.06	0.063
**Sex**								
male	Reference	.	.		Reference	.	.	
female	0.70	0.22	2.20	0.536	0.63	0.34	1.18	0.149
**Fur length**								
Shorthaired	Reference				Reference			
Longhaired	0.89	0.25	3.17	0.852	0.58	0.30	1.10	0.093
**Weight (kg)**								
<20	Reference	.	.		Reference	.	.	
≥20	1.66	0.50	5.58	0.406	1.13	0.63	2.04	0.684
**Age (years)**								
<6	Reference	.	.		Reference	.	.	
≥6	1.29	0.42	3.97	0.653	0.75	0.42	1.33	0.319
**Observer**								
neurologists	Reference	.	.		Reference	.	.	
practitioners	0.60	0.09	3.94	0.594	1.00	0.49	2.05	1.000
students	3.76	0.98	14.50	0.054	4.35	2.05	9.26	<0.001*

* = significant

### Reflex-activity

**Fig 1:**
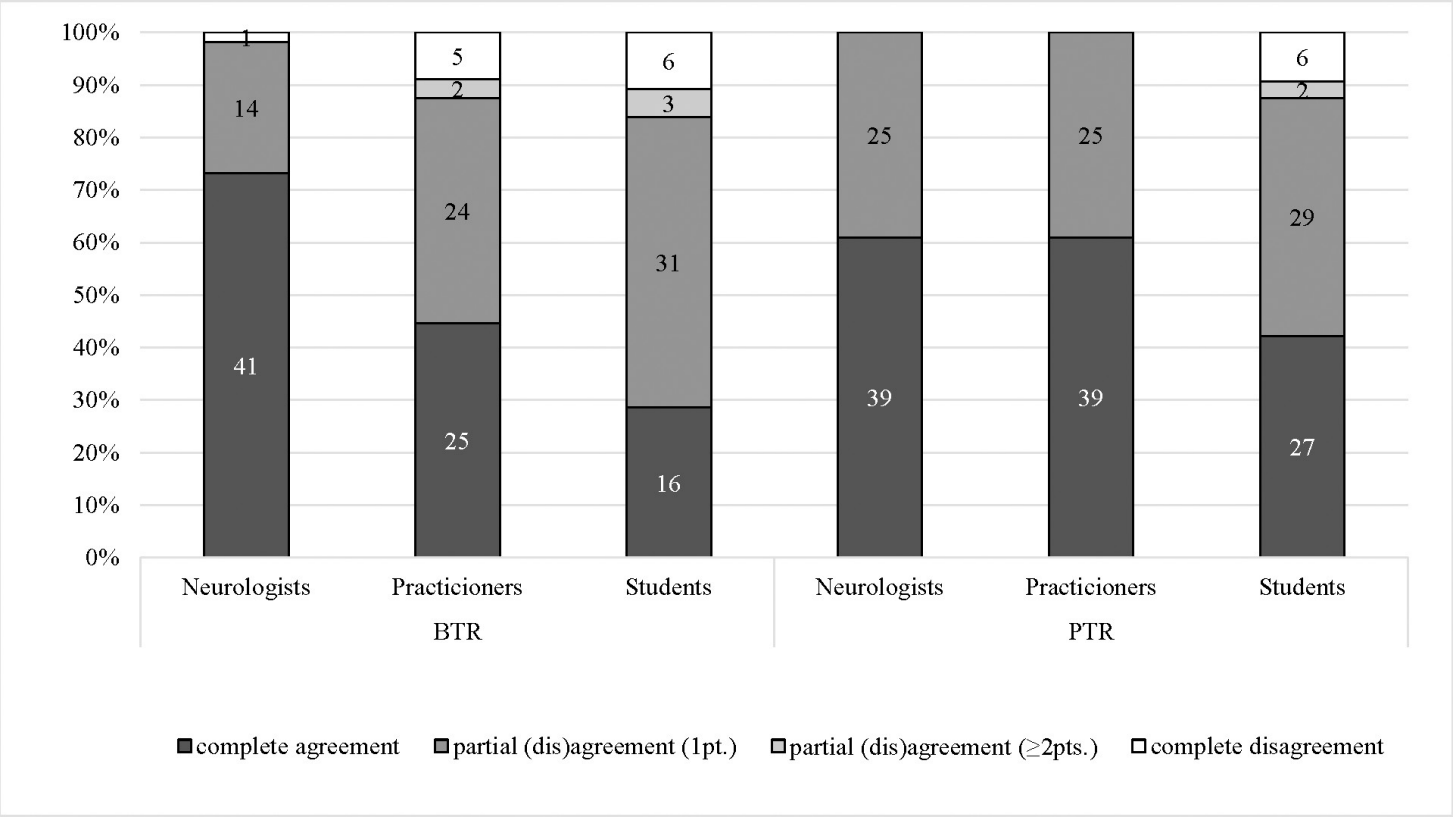
Agreement of reflex-activity. Subdivision of all evaluations for the biceps tendon reflex (BTR) and patellar tendon reflex (PTR) depending on their level of agreement: *complete agreement* was chosen for equal scoring by all three observers, *partial (dis)agreement* if one observer scored 1 point (1pt) or at least 2 points (≥ 2pts) higher or lower than the other two observers and *complete disagreement* if all observers scored differently.

All reliability coefficients are tabulated in Table 8. The IA of the reflex-activity (BTR) was significantly highest for neurologists than for practitioners (K_F Akt_: p = 0.022, ICC: p < 0.001) and students (K_F Akt_: p < 0.001, ICC: p < 0.001). The ICC (BTR) showed *questionable* reliability for the practitioners- and students-group, but *moderate* agreement for the neurologists (0.87; CI95%: 0.795–0.918). Kappa-analysis of reflex-activity (BTR) for all observer pairs could be interpreted as *clinically acceptable* for the neurologists, *inconclusive* for the practitioners and *clinically non-acceptable* for the students (S3 Table). The amount of *complete agreement*-evaluations increased with the level of the observer´s expertise (BTR) (Fig 1).

**Table 8:** Results of reliability analysis for the reflex activity.

	${\bar{X}}_{r\%}$	$\bar{X}K_{w}$	K_F Akt_	SE	CI95%		ICC	CI95%	
					lower	upper		lower	upper
**Biceps tendon reflex**									
**Neurologists**	81.6	0.57	0.49^a^	0.061	0.38	0.61	0.87^a^	0.80	0.92
**Practicioners**	60.7	0.44	0.37^b^	0.053	0.27	0.47	0.76^b^	0.63	0.85
**Students**	56.6	0.37	0.24^c^	0.048	0.15	0.34	0.76^b^	0.62	0.85
**Patellar tendon reflex**									
**Neurologists**	73.0	0.56	0.49^a^^,^^b^	0.057	0.38	0.60	0.85^a^^,^^b^	0.77	0.90
**Practicioners**	73.0	0.65	0.57^a^	0.051	0.47	0.67	0.90^a^	0.81	0.92
**Students**	58.3	0.54	0.43^b^	0.042	0.35	0.51	0.80^b^	0.76	0.90

${\bar{X}}_{r\%}$, mean percentage agreement between the three observer pairs of each group; $\bar{X}K_{w}$, mean K_w_ between the three observer pairs of each group

K_F Akt_, Fleiss´ Kappa for the reflex activity with its standard error (SE) and the lower and upper 95% confidence interval (CI95%) values; ICC, intraclass correlation coefficient with its CI95% values.

^a,b,c^, different letters indicate significant differences at p < 0.05.

Neurologists and practitioners had the identical distribution of *complete agreement*- and *partial disagreement*-evaluations of the reflex-activity (PTR) and showed no evaluation with a difference of more than one scale-point (Fig 1). Both groups showed a *moderate* agreement (ICC) for the PTR, while the students scored a *questionable* result. For neurologists and practitioners, kappa-statistics of reflex-activity (PTR) reached *clinically acceptable* results for each single pair of observers and was *clinically non-acceptable* in two cases in each group. For the student group, K_w_-interpretation was *clinically non-acceptable* for all the observer pairs (S4 Table).

Compared to each other, neurologists had a higher amount of *complete agreement*-evaluations for the BTR-evaluation than for the PTR, while practitioners and students had a higher amount of *complete agreement*-evaluations for the PTR. For practitioners and students, the $\bar{X}K_{w}$, $\bar{X}r\%$ and ICC were higher for the reflex-activity analysis of the PTR compared to the BTR, while neurologists scored more concordantly ($\bar{X}r\%$) the reflex-activity of the BTR or scored nearly equal for both reflexes ($\bar{X}r\%$, ICC). Regarding K_F Akt_, the reflex-activity (PTR) were evaluated significantly more consistent by practitioners and students than the reflex-activity (BTR) (both < 0.001). For practitioners this difference was significant for the ICC (p = 0.01) as well. For neurologists there was no difference between the IA of the reflex-activity (BTR) and reflex-activity (PTR).

For the reflex-activity (BTR), univariable regression analysis showed that only the *level of expertise* (p = 0.003; p < 0.001) and *fur length* (p = 0.003) significantly influenced the IA (Table 6). In longhaired dogs, the chance of disagreement was 2.6-times higher (p = 0.003) compared to shorthaired dogs in the evaluation of reflex-activity (BTR). In the multivariable regression (Table 9), practitioners had a 3.7-times (p = 0.002) and students a 7.9-times (p < 0.001) higher chance of discordance in judgements.

**Table 9:** Results of multivariable mixed regression analysis for the reflex activity (BTR) evaluation.

Risk factor	Reflex activity			
	Odds Ratio (OR)	CI 95% (OR)		p-value
		lower	upper	
**Fur length**				
shorthaired	Reference	.	.	.
longhaired	3.17	1.52	6.60	0.002*
**Observer**				
neurologists	Reference	.	.	
practitioners	3.74	1.62	8.62	0.002*
students	7.92	3.29	19.09	<0.001*

* = significant

The significant risk factors of the univariable mixed regression (Table 6) analysis are included.

For the reflex-activity (PTR), only the *observer´s level of expertise* had a significant (p < 0.001) impact on the IA. Students had a 4.4-times higher chance of discordance compared to the other groups. This effect was significant (p < 0.001). The chance of discordance is equal for practitioners and neurologists.

## Discussion

The evaluation of the reflex answer is considered to be an essential tool for the neurological examination despite its highly subjective nature. This study is the first to quantify and compare IA of the canine BTR and PTR and identifies possible risk factors for disagreement in clinical settings. The level of the observer´s expertise and the fur length of the dog had an impact on the degree of the IA of RA(BTR). The observer´s expertise had more of an influence on the evaluation of the bicipital tendon reflex than on the patellar tendon reflex.

Different authors have stated the impact that the level of observer experience has on the IA [7, 14] or discussed the improvement seen following training-sessions of the observers [16, 17, 30–33]. In this study, we opted not to train the observers prior to the evaluation in order to highlight the different IA dependant on the level of expertise. This study puts focus on a well-known problem in the daily clinical setting, where observers with a lower level of expertise must evaluate neurological patients during night shifts, interpret the findings and present them to specialists [34].

In many studies that focus on the IA of reflex evaluation, the examiner and the observer are the same person [19, 21, 35]. This study, however, has removed the influence that the level of expertise has in performing the procedures since the reflex examination was performed by the same individual, a doctoral student with a focus on clinical neurology with an expected level of expertise between group 1 and 2 (FG). It is therefore expected that these differing study designs would result in different findings regarding the reliability analysis presented here, but it remains unclear whether the approach taken in this study would result in a higher or lower IA.

Our results represent a widely discussed problem in medicine: the interobserver agreement of subjective evaluations [30, 36, 37]. The study design was influenced by existing veterinary and human medical literature. Levine et al. [11] let a blinded observer evaluate the reflex-presence of the canine PTR based on video-analysis. Stam and van Crevel [22] calculated the IA on video-analysis of different human spinal reflexes between three neurologists using a 9-point-ordinal scale. In addition, the inclusion criteria of this study were comparable to other studies that have examined the answer of different reflexes [38–40]. It is important to mention that we did not verify the integrity of the reflex arcs with an objective “gold-standard examination” such as magnetic resonance imaging and electromyography, as there is no “gold-standard” described. We only included clinically healthy dogs based on history and neurological examination. Therefore, the results could lack validity and this should be considered during interpretation of the results [7]. The high PI-values of both studies (S1 and S2 Tables) demonstrate that neurologists most often evaluate the reflex-answer as *normal*. A couple of studies in the veterinary and human medical literature have examined the IA of neurological symptoms based on video-analysis [7, 41–46]. The level of standardization varies heavily between these studies. With veterinary subjects, the standardization of a neurological examination is more difficult than with humans due to the lower compliance and the higher stress level of participants. There is, therefore, some limitation to the degree of standardization in this study which would otherwise not mimic the daily routine. The dogs analysed in this study were examined under clinical conditions in an identical manner, in the same room and using an identical set of tools. Nevertheless, the impact of quality of both video and examination on the IA cannot be quantified and it must be kept in mind, that the evaluation of standardized examination procedures based on video-analysis might result in an artificially high IA [43]. Considering the setting was the same for every observer, the results are comparable between the groups.

The interpretation of neurological signs via video-analysis is an emerging field of interest and already used in teleneurology in human medicine [34, 47, 48]. Telemedicine has also been introduced into veterinary medicine, but to the authors' knowledge, it has not been well established for neurology. Yager et al. [34] described a model in which the intensive care staff and a supervisor are able to communicate via video-conference during a night-shift. The intensive care staff presented three cases to the supervisor who through this medium was able to guide the stabilization of the patients. Our results show that both the BTR and PTR could be reliably assessed by neurologists using video-analysis.

Veterinary texts typically state that the PTR and withdrawal reflexes are thought to have the highest reliability [1, 3, 8]. However, various studies have questioned this idea. Forterre et al. [20] found that in nearly 30% of all examinations the withdrawal reflex of the forelimb was reduced although the myelopathy could be localized diagnostically within the spinal cord segments between the first and fifth cervical vertebrae. Murakami et al. [49] identified discrepancies in interpretation of the pelvic limb reflexes in dogs. They described the findings in dogs with confirmed lesions within the lower motor neuron reflex arc of the pelvic limb in which only 37.5% showed a reduced withdrawal reflex, and a reduced PTR was found only in 16.7%. Additionally, Abdelhakiem et al. [18] found in their study no lower motor neuron lesion to the pelvic limbs in dogs with a reduced reflex-activity (PTR). However, in nearly 30% of cases, a reduced reflex answer was misdiagnosed by the examiner for a lesion within the lower motor neuron of the pelvic limbs. It is also well established that the PTR must be interpreted with consideration of the age [11] and the position [50] of the patient.

In contrast to the work of Abdelhakiem et al. [18] and Forterre et al. [20], the patients in our study were all healthy and thus represented a homogenous group with high prevalence of the category 'normal'. Therefore, one could assume that the IA when assessing a reliable reflex should be 100%, however, perfect agreement is highly unlikely in medical studies [14]. In a more heterogeneous group including both normal dogs and dogs with lesions affecting the reflex arc of the BTR and/or PTR, a lower agreement would be expected. Nevertheless, it is important to clarify that assessing the accuracy of these reflexes in detecting a lesion within their reflex arc was not the aim of the study. In our opinion before being able to assess the reflex evaluation for its accuracy in detecting a lesion in the associated reflex arc, the IA and thereby its diagnostic utility has to be defined, especially since the evaluation of reflexes is based on subjective assessment [51, 52]. This study represents a logical consequence of the current subjective statements in the veterinary literature regarding the use of reflexes in the neurological evaluation of dogs, and this study provides baseline information on the assessment of reflex accuracy. Additionally, other studies that assessed the answer of different reflexes examined only healthy probands and thus our study design is comparable among the literature [11, 39, 40, 51, 52].

The presentation of multiple reliability coefficients depicts a trend of IA for each group and offers the possibility to interpret each coefficient in context to each other. Nevertheless, it is vital to recognize that each coefficient has its advantages and disadvantages. Therefore, the definition of the *clinical acceptance* for the interpretation of K-values under consideration of the percentage agreement by Sim and Wright [53] has been introduced into veterinary literature by Burn and Weir [29]. Regarding the results presented in this study, limitations of Kappa-statistics are obvious as there are two central paradoxes mentioned previously [53]. The first paradox is that Kappa might be low even there is a high percentage agreement, since percentage agreement is highly dependent on the prevalence of a category. The second paradox of Kappa-statistics states that an imbalanced and asymmetrical distribution of discordant evaluations (Bias) could result in a higher Kappa-value than in a balanced and symmetrical distribution.

A high prevalence of a category means a high homogeneity between the evaluations and thus an increase of the likelihood of an agreement just by chance. Burn and Weir [29] defined a pool of evaluations to be too homogenous if PI is > 0.90. In our study PI-values > 0.90 were only reached for the IA of the reflex-presence analysis and more often for the reflex-presence (PTR) than for reflex-presence (BTR). This results in a higher number of *clinically inconclusive* evaluations in the analysis of the IA of the reflex-presence of the PTR, as well as in K_C_-values ≤ 0.00. Paradox 2 means that a high BI might results in an artificially higher K-value. Like for the PI, there is no definition for the exact interpretation of the BI. The presented results show a few outliers with a relative higher BI (S4 Table). It could be assumed that the evaluations of the PTR were more homogenous than those of the BTR.

In clinical studies, Kappa mostly reaches values between 0.40–0.70, values between 0.60–0.80 are unusual and perfect agreement is highly unlikely [14]. The results of our study represent this distribution. K-values > 0.80 are restricted to neurologists and practitioners. Pairs of observers scored with K-values < 0.40 more often if their level of expertise was low (S2 and S3 Tables). In conclusion, K-values for each pair of observers presented here should be interpreted using the previously mentioned interpretation parameters PI, BI and K_max_ to distinguish between a poor IA and a statistical misinterpretation.

The IA is not comparable between different studies per se considering a difference in the study design including factors such as the number of observers or the categories of the ordinal-scale used. For example, in our study, we chose a very stringent model for the interpretation of the ICC [28]. Therefore, it can be assumed that the ICC would have been better interpreted when using the often-chosen model by Altman [54]. The interpretation of Kappa generally follows the model of Landis and Koch [27] and so its values are comparable between the studies with the consideration of the respective study design.

For humans it has been shown, that reflex-activity of the PTR scored higher with extent of the knee-angle changing and decreasing reflex-time [7]. Our study design does not allow an identification of equivalent parameter, however, we identified two risk factors that increased the likelihood of discordant evaluations. Thomas and Dewey [24] already assumed a difficulty in the correct interpretation of the canine BTR in dogs with long fur. Our results show a significant increase in discordance for reflex-activity (BTR) in longhaired dogs. This effect could not be observed for the reflex-activity (PTR). It can be postulated that the visibility of the flexion of the elbow or the contraction of the biceps brachii, might be more affected by long fur than the extension of the stifle joint. Additionally, the level of the observer`s expertise has a higher impact on the IA of reflex-activity (BTR) than on the reflex-activity (PTR). Since descriptions of the evaluation of the BTR are generally limited to veterinary neurology literature, it is expected that its interpretation is limited to more specialized observers. In contrast to this, the PTR is the typical and well-known monosynaptic reflex and thus its reflex answer will be more familiar to and therefore more often correctly interpreted by observers even with a lower level of expertise. Following our results, it could be concluded that the reflex answer of the BTR interpreted by an examiner with a lower level of expertise should be considered with caution, whilst the PTR is more reliable between examiners with a different level of expertise.

Objectification of the neurological examination is a major topic of current veterinary research [15, 18, 20, 45, 46, 51, 52, 55] with increasing recognition of the importance of evaluating the reliability and therefore the utility of different neurological examination parameters. Our results highlight the need to objectively evaluate the neurological examination and to consider the many factors that might influence its assessment and therefore decrease its reliability.

## Conclusions

The BTR could be reliably assessed by veterinary neurologists. The interpretation of the reflex answer of the BTR is more vulnerable to the level of the observer`s expertise and the fur length of the dog than the interpretation of the PTR. Neurologists are able to evaluate the BTR and the PTR reliably even via video-analysis. The study design presented here could serve as a model for the potential use of teleneurology in veterinary medicine.

## Supporting information

S1 TableResults of reliability analysis for the reflex presence (BTR).Note that all reliability coefficients are the higher the higher the level of the observers´ expertise is. r%, percentage agreement; ${\bar{X}}_{r\%}$, mean percentage agreement between the three observer pairs of each group; K_C_, Cohen´s Kappa; CA, category of clinical acceptance with I, clinically acceptable, II, clinically non-acceptable, III, inconclusive; PI, Prevalence-Index; BI, Bias-Index; K_max_, maximum Kappa; $\bar{X}$ K_C_, mean K_C_ between the three observer pairs of each group; K_F Pres_, Fleiss´ Kappa with its standard error (SE) and the lower and upper 95% confidence interval (CI95%) values; ICC, intraclass correlation coefficient with its CI95% values. ^a,b,c^, different letters indicate significant differences at p < 0.05.(DOCX)

S2 TableResults of reliability analysis for the reflex presence (PTR).Note that ICC and K_F_ are the higher the higher the level of observer´s expertise is. Note the relatively high number of inclonclusive evaluations due to a low. r%, percentage agreement; ${\bar{X}}_{r\%}$, mean percentage agreement between the three observer pairs of each group; K_C_, Cohen´s Kappa; CA, category of clinical acceptance with I, clinically acceptable, II, clinically non-acceptable, III, inconclusive; PI, Prevalence-Index; BI, Bias-Index; K_max_, maximum Kappa; $\bar{X}$ K_C_, mean K_C_ between the three observer pairs of each group; K_F Pres_, Fleiss´ Kappa with its standard error (SE) and the lower and upper 95% confidence interval (CI95%) values; ICC, intraclass correlation coefficient with its CI95% values. ^a,b^, different letters indicate significant differences at p < 0.05.(DOCX)

S3 TableResults of reliability analysis for the reflex activity (BTR).Note that clinical acceptance is the more acceptable the higher the level of the observer´s expertise is. r%, percentage agreement; ${\bar{X}}_{r\%}$, mean percentage agreement between the three observer pairs of each group; K_w_, weighted Kappa; CA, category of clinical acceptance with I, clinically acceptable, II, clinically non-acceptable, III, inconclusive; PI, Prevalence-Index; BI, Bias-Index; K_max_, maximum Kappa; $\bar{X}$ K_w_, mean K_w_ between the three observer pairs of each group; K_F Akt_, Fleiss´ Kappa with its standard error (SE) and the lower and upper 95% confidence interval (CI95%) values; ICC, intraclass correlation coefficient with its CI95% values. ^a,b,c^, different letters indicate significant differences at p < 0.05.(DOCX)

S4 TableResults of reliability analysis for the reflex activity (PTR).Note the high number of clinically non-acceptable evaluations in all groups. r%, percentage agreement; ${\bar{X}}_{r\%}$, mean percentage agreement between the three observer pairs of each group; K_w_, weighted Kappa; CA, category of clinical acceptance with I, clinically acceptable, II, clinically non-acceptable, III, inconclusive; PI, Prevalence-Index; BI, Bias-Index; K_max_, maximum Kappa; $\bar{X}$ K_w_, mean K_w_ between the three observer pairs of each group; K_F Akt_, Fleiss´ Kappa with its standard error (SE) and the lower and upper 95% confidence interval (CI95%) values; ICC, intraclass correlation coefficient with its CI95% values. ^a,b^, different letters indicate significant differences at p < 0.05.(DOCX)
