# Supplementary material for: Comparison of interobserver agreement between the evaluation of bicipital and the patellar tendon reflex in healthy dogs
Source: PLoS One. 2019 Jul 10;14(7):e0219171. doi: 10.1371/journal.pone.0219171 (PMC6619687; doi:10.1371/journal.pone.0219171)
Supplement: S1 Table — Note that all reliability coefficients are the higher the higher the level of the observers´ expertise is. r%, percentage agreement; X¯r%, mean percentage agreement between the three observer pairs of each group; KC, Cohen´s Kappa; CA, category of clinical acceptance with I, clinically acceptable, II, clinically non-acceptable, III, inconclusive; PI, Prevalence-Index; BI, Bias-Index; Kmax, maximum Kappa; X¯ KC, mean KC between the three observer pairs of each group; KF Pres, Fleiss´ Kappa with its standard error (SE) and the lower and upper 95% confidence interval (CI95%) values; ICC, intraclass correlation coefficient with its CI95% values. a,b,c, different letters indicate significant differences at p < 0.05. (DOCX) [file pone.0219171.s001.docx]

|  | **r%** | **X̅_r%_** | **K_C_** | **CA** | **PI** | **BI** | **K_max_** | **X̅K_C_** | **K_F Pres_** | **SE** | **CI95%** | | **ICC** | **CI95%** | |
| --- | --- | --- | --- | --- | --- | --- | --- | --- | --- | --- | --- | --- | --- | --- | --- |
|  |  |  |  |  |  |  |  |  |  |  | **lower** | **upper** |  | **lower** | **upper** |
| **Neurologists** | | | | | | | | | | | | | | | |
| N1-N3 | 98.2 | 97.6 | 0.79 | I | 0.91 | 0.00 | 0.98 | 0.76 | 0.77^a^ | 0.077 | 0.61 | 0.92 | 0.91^a^ | 0.86 | 0.94 |
| N1-N2 | 96.4 |  | 0.65 | I | 0.89 | 0.04 | 0.96 |  |  |  |  |  |  |  |  |
| N2-N3 | 98.2 |  | 0.85 | I | 0.88 | 0.02 | 0.98 |  |  |  |  |  |  |  |  |
| **Practitioners** | | | | | | | | | | | | | | | |
| P1-P3 | 92.9 | 95.2 | 0.47 | I | 0.86 | 0.07 | 0.47 | 0.63 | 0.64^a^ | 0.077 | 0.49 | 0.79 | 0.85^b^ | 0.77 | 0.91 |
| P1-P2 | 96.4 |  | 0.65 | I | 0.89 | 0.04 | 0.65 |  |  |  |  |  |  |  |  |
| P2-P3 | 96.4 |  | 0.78 | I | 0.82 | 0.04 | 0.78 |  |  |  |  |  |  |  |  |
| **Students** | | | | | | | | | | | | | | | |
| S1-S3 | 94.6 | 91.7 | 0.55 | I | 0.88 | 0.02 | 0.55 | 0.47 | 0.45^b^ | 0.077 | 0.30 | 0.61 | 0.73^c^ | 0.58 | 0.83 |
| S1-S2 | 91.1 |  | 0.41 | I | 0.84 | 0.09 | 0.41 |  |  |  |  |  |  |  |  |
| S2-S3 | 89.3 |  | 0.44 | I | 0.79 | 0.04 | 0.81 |  |  |  |  |  |  |  |  |
