# Supplementary material for: Comparison of interobserver agreement between the evaluation of bicipital and the patellar tendon reflex in healthy dogs
Source: PLoS One. 2019 Jul 10;14(7):e0219171. doi: 10.1371/journal.pone.0219171 (PMC6619687; doi:10.1371/journal.pone.0219171)
Supplement: S2 Table — Note that ICC and KF are the higher the higher the level of observer´s expertise is. Note the relatively high number of inclonclusive evaluations due to a low. r%, percentage agreement; X¯r%, mean percentage agreement between the three observer pairs of each group; KC, Cohen´s Kappa; CA, category of clinical acceptance with I, clinically acceptable, II, clinically non-acceptable, III, inconclusive; PI, Prevalence-Index; BI, Bias-Index; Kmax, maximum Kappa; X¯ KC, mean KC between the three observer pairs of each group; KF Pres, Fleiss´ Kappa with its standard error (SE) and the lower and upper 95% confidence interval (CI95%) values; ICC, intraclass correlation coefficient with its CI95% values. a,b, different letters indicate significant differences at p < 0.05. (DOCX) [file pone.0219171.s002.docx]

|  | **r%** | **X̅_r%_** | **K_C_** | **CA** | **PI** | **BI** | **K_max_** | **X̅K_C_** | **K_F Pres_** | **SE** | **CI95%** | | **ICC** | **CI95%** | |
| --- | --- | --- | --- | --- | --- | --- | --- | --- | --- | --- | --- | --- | --- | --- | --- |
|  |  |  |  |  |  |  |  |  |  |  | **lower** | **upper** |  | **lower** | **upper** |
| **Neurologists** | | | | | | | | | | | | | | | |
| N1-N3 | 96.9 | 97.9 | -0.02 | III | 0.96 | 0.00 | 1.00 | 0.43 | 0.49^a^ | 0.072 | 0.35 | 0.63 | 0.74^a^ | 0.61 | 0.84 |
| N1-N2 | 98.4 |  | 0.66 | I | 0.95 | 0.02 | 0.66 |  |  |  |  |  |  |  |  |
| N2-N3 | 98.4 |  | 0.66 | I | 0.66 | 0.02 | 0.66 |  |  |  |  |  |  |  |  |
| **Practitioners** | | | | | | | | | | | | | | | |
| P1-P3 | 96.9 | 97.9 | 0.00 | III | 0.97 | 0.03 | 0.00 | 0.22 | 0.32^b^ | 0.072 | 0.18 | 0.46 | 0.60^a,b^ | 0.39 | 0.74 |
| P1-P2 | 98.4 |  | 0.66 | I | 0.95 | 0.02 | 0.66 |  |  |  |  |  |  |  |  |
| P2-P3 | 98.4 |  | 0.00 | III | 0.98 | 0.02 | 0.00 |  |  |  |  |  |  |  |  |
| **Students** | | | | | | | | | | | | | | | |
| S1-S3 | 93.8 | 89.6 | 0.32 | III | 0.91 | 0.06 | 0.32 | 0.25 | 0.23^b^ | 0.072 | 0.09 | 0.37 | 0.50^b^ | 0.25 | 0.68 |
| S1-S2 | 89.1 |  | 0.20 | III | 0.86 | 0.12 | 0.20 |  |  |  |  |  |  |  |  |
| S2-S3 | 85.9 |  | 0.23 | III | 0.80 | 0.05 | 0.74 |  |  |  |  |  |  |  |  |
