# Supplementary material for: Comparison of interobserver agreement between the evaluation of bicipital and the patellar tendon reflex in healthy dogs
Source: PLoS One. 2019 Jul 10;14(7):e0219171. doi: 10.1371/journal.pone.0219171 (PMC6619687; doi:10.1371/journal.pone.0219171)
Supplement: S3 Table — Note that clinical acceptance is the more acceptable the higher the level of the observer´s expertise is. r%, percentage agreement; X¯r%, mean percentage agreement between the three observer pairs of each group; Kw, weighted Kappa; CA, category of clinical acceptance with I, clinically acceptable, II, clinically non-acceptable, III, inconclusive; PI, Prevalence-Index; BI, Bias-Index; Kmax, maximum Kappa; X¯ Kw, mean Kw between the three observer pairs of each group; KF Akt, Fleiss´ Kappa with its standard error (SE) and the lower and upper 95% confidence interval (CI95%) values; ICC, intraclass correlation coefficient with its CI95% values. a,b,c, different letters indicate significant differences at p < 0.05. (DOCX) [file pone.0219171.s003.docx]

|  | **r%** | **X̅_r%_** | **K_w_** | **CA** | **PI** | **BI** | **K_max_** | **X̅K_w_** | **K_F Akt_** | **SE** | **CI95%** | | **ICC** | **CI95%** | |
| --- | --- | --- | --- | --- | --- | --- | --- | --- | --- | --- | --- | --- | --- | --- | --- |
|  |  |  |  |  |  |  |  |  |  |  | **lower** | **upper** |  | **lower** | **upper** |
| **Neurologists** | | | | | | | | | | | | | | | |
| N1-N3 | 80.4 | 81.6 | 0.55 | I | 0.31 | 0.04 | 0.85 | 0.57 | 0.49^a^ | 0.061 | 0.38 | 0.61 | 0.87^a^ | 0.80 | 0.92 |
| N1-N2 | 75.0 |  | 0.43 | I | 0.31 | 0.04 | 0.90 |  |  |  |  |  |  |  |  |
| N2-N3 | 89.3 |  | 0.74 | I | 0.29 | 0.00 | 0.78 |  |  |  |  |  |  |  |  |
| **Practitioners** | | | | | | | | | | | | | | | |
| P1-P3 | 67.9 | 60.7 | 0.47 | II | 0.04 | 0.10 | 0.70 | 0.44 | 0.37^b^ | 0.053 | 0.27 | 0.47 | 0.76^b^ | 0.63 | 0.85 |
| P1-P2 | 55.4 |  | 0.41 | II | 0.04 | 0.11 | 0.91 |  |  |  |  |  |  |  |  |
| P2-P3 | 58.9 |  | 0.43 | II | 0.02 | 0.00 | 0.70 |  |  |  |  |  |  |  |  |
| **Students** | | | | | | | | | | | | | | | |
| S1-S3 | 62.5 | 56.6 | 0.38 | III | 0.00 | 0.18 | 0.65 | 0.37 | 0.24^c^ | 0.048 | 0.15 | 0.34 | 0.76^b^ | 0.62 | 0.85 |
| S1-S2 | 55.4 |  | 0.33 | III | 0.00 | 0.18 | 0.81 |  |  |  |  |  |  |  |  |
| S2-S3 | 51.8 |  | 0.41 | III | 0.10 | 0.00 | 0.90 |  |  |  |  |  |  |  |  |
