# Supplementary material for: Comparison of interobserver agreement between the evaluation of bicipital and the patellar tendon reflex in healthy dogs
Source: PLoS One. 2019 Jul 10;14(7):e0219171. doi: 10.1371/journal.pone.0219171 (PMC6619687; doi:10.1371/journal.pone.0219171)
Supplement: S4 Table — Note the high number of clinically non-acceptable evaluations in all groups. r%, percentage agreement; X¯r%, mean percentage agreement between the three observer pairs of each group; Kw, weighted Kappa; CA, category of clinical acceptance with I, clinically acceptable, II, clinically non-acceptable, III, inconclusive; PI, Prevalence-Index; BI, Bias-Index; Kmax, maximum Kappa; X¯ Kw, mean Kw between the three observer pairs of each group; KF Akt, Fleiss´ Kappa with its standard error (SE) and the lower and upper 95% confidence interval (CI95%) values; ICC, intraclass correlation coefficient with its CI95% values. a,b, different letters indicate significant differences at p < 0.05. (DOCX) [file pone.0219171.s004.docx]

|  | **r%** | **X̅_r%_** | **K_w_** | **CA** | **PI** | **BI** | **K_max_** | **X̅K_w_** | **K_F_** | **SE** | **CI95%** | | **ICC** | **CI95%** | |
| --- | --- | --- | --- | --- | --- | --- | --- | --- | --- | --- | --- | --- | --- | --- | --- |
|  |  |  |  |  |  |  |  |  |  |  | **lower** | **upper** |  | **lower** | **upper** |
| **Neurologists** | | | | | | | | | | | | | | | |
| N1-N3 | 68.6 | 74.0 | 0.46 | II | 0.14 | 0.09 | 0.85 | 0.56 | 0.49^a,b^ | 0.057 | 0.38 | 0.60 | 0.85^a,b^ | 0.77 | 0.90 |
| N1-N2 | 84.4 |  | 0.72 | I | 0.22 | 0.05 | 0.87 |  |  |  |  |  |  |  |  |
| N2-N3 | 68.8 |  | 0.49 | II | 0.17 | 0.14 | 0.68 |  |  |  |  |  |  |  |  |
| **Practitioners** | | | | | | | | | | | | | | | |
| P1-P3 | 67.2 | 74.0 | 0.57 | II | 0.03 | 0.27 | 0.59 | 0.65 | 0.57^a^ | 0.051 | 0.47 | 0.67 | 0.90^a^ | 0.81 | 0.92 |
| P1-P2 | 70.3 |  | 0.64 | II | 0.02 | 0.19 | 0.71 |  |  |  |  |  |  |  |  |
| P2-P3 | 84.4 |  | 0.75 | I | 0.15 | 0.08 | 0.86 |  |  |  |  |  |  |  |  |
| **Students** | | | | | | | | | | | | | | | |
| S1-S3 | 62.5 | 58.3 | 0.53 | II | 0.12 | 0.09 | 0.89 | 0.54 | 0.43^b^ | 0.042 | 0.35 | 0.51 | 0.80^b^ | 0.76 | 0.90 |
| S1-S2 | 57.8 |  | 0.54 | II | 0.18 | 0.20 | 0.81 |  |  |  |  |  |  |  |  |
| S2-S3 | 54.7 |  | 0.56 | II | 0.24 | 0.11 | 0.86 |  |  |  |  |  |  |  |  |
